# Controlling CNT-Based Nanorotors via Hydroxyl Groups

**DOI:** 10.3390/nano12193363

**Published:** 2022-09-27

**Authors:** Boyang Zhang, Rui Li, Qing Peng

**Affiliations:** 1 School of Mechanical Engineering, University of Science and Technology Beijing, Beijing 100083, China; 2 Physics Department, King Fahd University of Petroleum and Minerals, Dhahran 31261, Saudi Arabia; 3 K.A.CARE Energy Research and Innovation Center at Dhahran, Dhahran 31261, Saudi Arabia; 4 Interdisciplinary Research Center for Hydrogen and Energy Storage, King Fahd University of Petroleum and Minerals, Dhahran 31261, Saudi Arabia

**Keywords:** transmission system, carbon nanotube (CNT), hydroxyl groups, response speed, energy dissipation

## Abstract

Nanomotor systems have attracted extensive attention due to their applications in nanorobots and nanodevices. The control of their response is crucial but presents a great challenge. In this work, the rotating and braking processes of a carbon nanotube (CNT)-based rotor system have been studied using molecular dynamics simulation. The speed of response can be tuned by controlling the ratio of hydroxyl groups on the edges. The ratio of hydroxyl groups is positively correlated with the speed of response. The mechanism involved is that the strong hydrogen bonds formed between interfaces increase the interface interaction. Incremental increase in the hydroxyl group concentration causes more hydrogen bonds and thus strengthens the interconnection, resulting in the enhancement of the speed of response. The phonon density of states analysis reveals that the vibration of hydroxyl groups plays the key role in energy dissipation. Our results suggest a novel routine to remotely control the nanomotors by modulating the chemical environment, including tuning the hydroxyl groups concentration and pH chemistry.

## 1. Introduction

With the development of nanotechnology, nano machines and nano devices have attracted extensive attention. Many nano machines such as molecular car motors [1], elevators [2] and shuttles [3] have been designed. Carbon nanotubes are one of the most important candidates in developing micro-electromechanical and nano-mechanical systems owing to their excellent mechanical characters, unique structures, high flexibility, and super-lubrication between multi-walled carbon nanotubes. Multi-walled carbon tubes have been applied to design nano tweezers [4,5], nano gears [6], gigahertz oscillators [7,8,9], nano bearings [10,11,12,13], nano motors [14,15,16,17], and nano bump [18].

Nano transmission systems that transfer motion and energies based on carbon nanotubes have also attracted extensive attention. There are two main kinds of transmission systems. One is to investigate the relative movement of inner and outer tubes in the axial direction. Barreiro et al. [19] studied the relative movement of the short outer tube relative to the long inner tube of multi-walled carbon nanotubes under axial thermal gradient. Santamaría-Holek et al. [20] proposed a model combining the actions of friction, van der Waals, and thermal forces and the effects of noise to explain the motion of a carbon nanotube along the other coaxial carbon nanotube. Another approach has been to design a nano-rotation transmission system using the interface interaction. Cai et al. [21,22] combined the carbon nanotube motor with a multi-walled carbon nanotube bearing to form a transmission system. Based on this design, Qiu et al. [23] developed a multi-level transmission system. Yin et al. [24,25], Gao et al. [26], Zhang et al. [7], Song et al. [27], Shi et al. [28] also studied transmission systems following a similar design. The above research all focused on using the interaction between hydrogen groups to achieve transmission. The hydrogen bond formed between hydroxyl groups could increase the interface interaction [29,30]. Our previous work [31] showed that hydroxyl groups could enhance the transmission efficiency owing to the strengthening of the interaction. We speculate that hydroxyl groups might also enhance the response of the transmission system. Therefore, in this work, we have investigated the acceleration, braking process and energy dissipation of the transmission system based on double-walled carbon nanotubes grafted with hydroxyl groups via molecular dynamics simulation. The effect of hydroxyl groups on the response of the system is evaluated.

## 2. Model and Method

**Figure 1:**
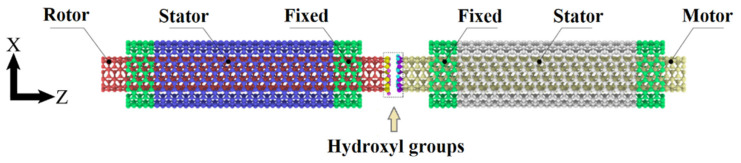
Illustration of the transmission system consisting of two DWCNTs.

The model of the transmission system is shown in Figure 1. The system includes two identical double-walled carbon nanotubes, which are the motor on the left and the rotor on the right, respectively. The double-walled carbon nanotubes (DWCNT) are applied, which include SWCNT (5, 5) and SWCNT (10, 10). Their diameters are 0.69 and 1.38 nm, respectively. Both ends of the outer tube SWCNT (10, 10) are fixed to avoid movement. Hydroxyl groups are grafted on the end of the inner tube between interfaces. The number of hydroxyl groups to the number of C atoms on the corresponding ends is defined as the hydroxyl group ratio. The length of inner and outer tube is 5.90 and 4.91 nm, respectively. Zhu et al. [12] pointed out that the energy dissipation between tubes in DWCNT was approximately proportional to the contact area. Changing the length of carbon nanotubes does not influence the energy dissipation rate. Therefore, the length of carbon nanotubes is kept the same during simulations.

The interaction among the C atoms of carbon nanotubes is described by AIREBO [32]. An OPLS_AA force field [33,34] is applied to describe C-O-H on the end of the inner carbon nanotube. Van der Waals force between interfaces is described by the 12-6 Lennard-Jones potential [35]. A DREIDING field [36] is applied to calculate the hydrogen bond between motor and rotor. The MD time step is 0.001 ps. This value is carefully selected as a compromise between numerical stability and computing resources. This value is also commonly adopted and successfully applied in similar transmission systems based on carbon nanotubes [23]. The Nose-Hoover method is applied to keep the temperature at 300 K. 

The simulation process has three stages. At first, the whole system is relaxed for 200 ps. At the second stage, the four layers of atoms on the left end of the motor rotate at a constant frequency. The rotor on the right also begins to rotate because of the interaction between interfaces. At the last stage, the motion of the motor is removed to simulate the deceleration process. Consequently, the rotor gradually slows down to stop.

## 3. Results and Discussion

### 3.1. Transmission

**Figure 2:**
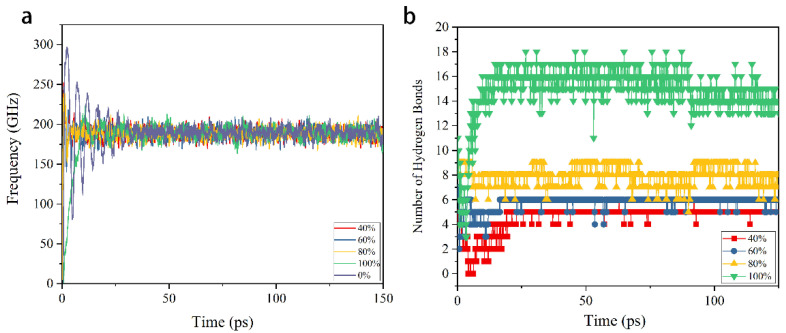
(**a**) The rotation frequency of rotor when pristine CNTs, CNTs with 40%, 60%, 80%, 100% grafted hydroxyl groups are applied. (**b**) The hydrogen bonds in five cases.

Owing to the interaction between interfaces, the rotor begins to rotate when a constant speed is applied to the left-end of the motor. Figure 2a shows the rotation frequency of the rotor in five cases as pristine DWCNTs, the interface grafted with 40%, 60%, 80% and 100% hydroxyl groups. The results show that the rotor reaches a stable state with rotation frequency 200 GHz in about 30 ps in all cases, which is consistent with the rotation frequency of the motor. The maximum amplitude of vibration occurs in the case with original DWCNTs, a phenomenon that can be attributed to the interaction between interfaces [31]. When pristine carbon nanotubes are applied, the interaction between interfaces is only the van der Waals force. However, hydrogen bonds form between interfaces in other cases, as shown in Figure 2b. The higher the hydroxyl group ratio is, the larger the number of hydrogen bonds that form.

### 3.2. Braking Process

**Figure 3:**
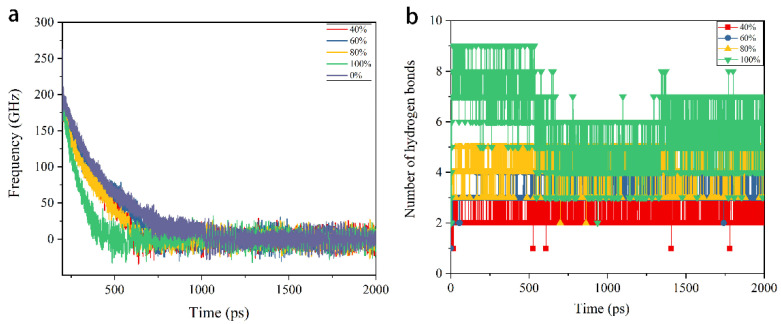
(**a**) The rotation frequency of rotor during braking process when pristine CNTs, CNTs with 40%, 60%, 80%, 100% grafted hydroxyl groups are applied. (**b**) The number of hydrogen bonds when 40%, 60%, 80%, 100% hydroxyl groups are grafted, respectively.

The braking process starts from 200 ps when the motion is removed from the motor. Owing to the interaction between interfaces, the rotor gradually decelerates. The rotation frequency of the rotor during the deceleration process is shown in Figure 3a, which includes the cases with pristine carbon nanotubes, with 40%, 60%, 80%, and 100% grafted hydroxyl groups on the interface. For the case with pristine DWCNTs, the rotor stops rotation at about 1000 ps. The rotor with higher hydroxyl groups stops earlier due to the stronger interaction between interfaces. More hydrogen bonds form when higher ratios of hydroxyl groups are grafted, as shown in Figure 3b. The stability of transmission systems during the braking process are also examined. The results show that the vibration of the centroid of the rotor in the x direction is below 0.04 nm, which implies that the rotor stabilizes in all cases.

### 3.3. Energy Dissipation

**Figure 4:**
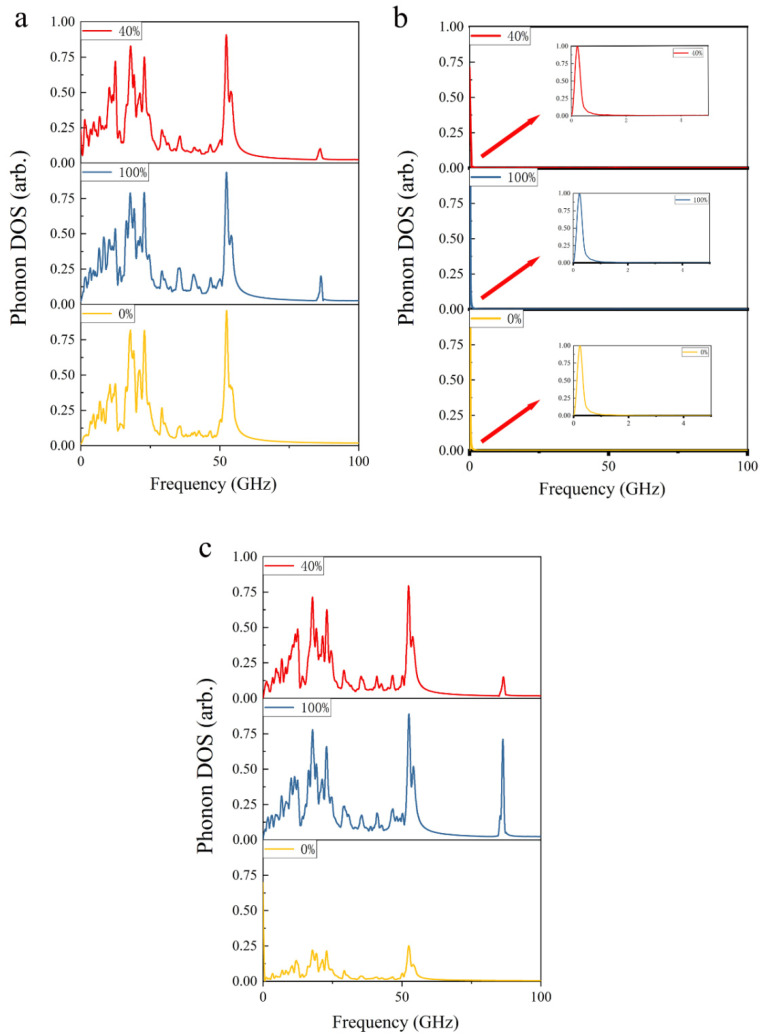
The phonon DOS of the transmission system in three stages when pristine carbon nanotubes, carbon nanotubes grafted with 40% and 100% hydroxyl groups are applied, respectively: (**a**) relaxation, (**b**) acceleration, and (**c**) deceleration.

To further explore the energy dissipation behavior of the transmission system during the whole process, the phonon density of state (DOS) of the rotor are calculated in three cases namely pristine carbon nanotubes, and those grafted with 40% and 100% hydroxyl groups, respectively, as shown in Figure 4. The subfigures (a–c) are in relaxation stage, 200–250 ps of rotation stage and 400~450 ps of deceleration stage, respectively. Konstantin [37] pointed out that that multi-walled carbon nanotubes generally have radial breathing mode (RBM), D band, and G band, where D and G bands represent the defects and in-plane stretching vibration of carbon nanotubes. The peaks of D band and G band in Raman spectra are at 150 cm^−1^ (inner tube), 300 cm^−1^ (outer tube), 1350 cm^−1^ and 1582 cm^−1^, respectively. The peaks of hydroxyl groups in Raman spectra mainly include the vibration of C-O bond, O-H bond and out-of-plane bending vibration of O-H bonds, which are at 3200 cm^−1^, 1200 cm^−1^ and 660 cm^−1^, respectively. According to the equation k = f/c, where k, f and c are the wave number, the frequency, and the speed of light, respectively. Therefore, the frequencies of RBM, D band and G band of the multi-walled carbon nanotube are 4.50 (inner tube) and 9.00 (outer tube), 40.50 and 47.46 THz. The three peaks of the hydroxyl group are at 19.80, 36.00 and 86.00 THz, respectively. 

In the relaxation stage, as shown in Figure 4a, the peaks of Phonon DOS of the system are similar in all three cases. Compared to pristine carbon nanotube, the most distinct difference in the cases with hydroxyl groups is the peak position at 86 THz. This clearly implies that the O-H bond vibrates in the out-of-plane direction. The other peaks represent RBMs, 2RBMs and G band with a slight frequency shift. Owing to the ideal carbon nanotubes applied in simulation, the D band is not obvious in the case with original carbon nanotubes, whereas it can be observed in the case grafted with 100% hydroxyl group. In the rotation stage, there is no peak in phonon DOS of the system because the carbon nanotube is in high constant speed rotation. During the deceleration stage at 400–450 ps, the phonon DOS is similar to the relaxation stage. The vibration of carbon nanotubes is weakened, although the out-of-plane bending vibration of O-H bonds is more obvious. Our results show that the vibration of O-H bonds accelerates the energy dissipation of the system and leads to a faster braking process.

## 4. Conclusions

The rotating and braking processes of a carbon nanotube transmission system have been investigated via molecular dynamics simulations. The effect of hydroxyl groups on the speed of response is examined. The energy dissipation during the whole process is discussed.

The results show that hydroxyl groups enhance the stability and reduce the response time of the system in both the acceleration and braking process. The higher the hydroxyl group ratio is used, the better the performance of the transmission system achieved. The underlying mechanism is the presence of hydrogen bonds that form between hydroxyl groups. These hydrogen bonds result in higher interface interaction and a faster response.

The analysis of the phonon density of state shows that the vibration of O-H bonds in hydroxyl groups accelerates energy dissipation of the system, which leads to faster response in the acceleration and braking process. Our results show that grafted hydroxyl groups result in stronger interaction, and therefore have potential in enhancing the response of the transmission system.

## Data Availability

Data available on request.
